# MSC - targets for atherosclerosis therapy

**DOI:** 10.18632/aging.101735

**Published:** 2018-12-26

**Authors:** Ines Colmegna, Ursula Stochaj

**Affiliations:** 1 Division of Rheumatology, Department of Medicine, McGill University, Montreal, QC, Canada; 2 Department of Physiology, McGill University, Montreal, QC, Canada

**Keywords:** mesenchymal stromal cells, atherosclerosis, mitochondria, aging

Atherosclerosis, a chronic inflammatory disease of the wall of large- and medium-sized arteries, is the most common pathological process leading to cardiovascular disease (CVD). The hallmark lesion in atherosclerosis is the atherosclerotic plaque. Immune dysregulation and inflammation are key contributors to the development of an atherosclerotic plaque and its progression to acute coronary syndromes. Atherosclerotic plaques consist of necrotic cores, calcified regions, modified lipids, and predominantly inflammatory cell infiltrates. All phases of atherosclerosis are regulated by inflammatory mechanisms which affect immune cell function, endothelial activation, and metabolic parameters [1]. Recognition of the pivotal contribution of inflammation in atherosclerosis opened up a new treatment paradigm: the possibility to mitigate CVD burden through immune modulation [2]. Therapies targeting inflammatory pathways (e.g. pro-inflammatory cytokine inhibitors, anti-metabolites, leukotriene inhibitors) are currently under investigation in pre-clinical and clinical studies.

An alternative strategy to target inflammatory pathways for CVD therapy could be enhancing physiological mechanisms that antagonize inflammation. Key cellular targets for this approach are multipotent mesenchymal stromal cells (MSC). MSC are non-hematopoietic clonogenic perivascular multipotent stromal cells that can be induced to differentiate *in vitro* into osteoblasts, chrondrocytes or adipocytes. MSC function as pivotal regulators of inflammation by modulating innate and adaptive immune cells. This does not require long-term engraftment of MSC in target tissues [3]. The crosstalk between MSC and immune cells is mainly mediated by secreted bioactive molecules (e.g. cytokines, growth factors, anti-inflammatory mediators), and extracellular vesicles, which are also present in the MSC secretome. The balance between anti- and pro-inflammatory factors in the MSC secretome determines the MSC immunopotency. Of relevance, the immunomodulatory capabilities of MSC are not constitutive and require MSC to be primed (i.e. activated) by inflammatory cytokines.

Experimental models of atherosclerosis confirmed the atheroprotective effect of MSC treatment. The following mechanisms contribute to MSC-dependent atheroprotection: (1) the production of anti-inflammatory factors (e.g. TSG-6, IL-10), (2) NFκB and MMP inhibition in the plaque, (3) the modulation of the cellular plaque composition (e.g. increase of intralesional T-regs), (4) the repair of endothelial damage, and (5) the reduction of the number of apoptotic cells in the tunica media, intima and lipid core [4]. Together, these five processes promote plaque stability. Factors present in the MSC secretome mediate all of these mechanisms.

Limited data are available for MSC from patients with atherosclerosis. Specifically, the contribution of MSC dysfunction to the persistence of chronic inflammation and plaque progression are ill-defined. This relates in part to the lack of specific markers that can identify MSC *in vivo* in human arteries. We have overcome this obstacle by using an alternative approach. Thus, we have characterized adipose derived MSC from atherosclerotic patients (i.e. subjects undergoing coronary artery bypass graft surgery) and compared their function with MSC from non-atherosclerotic patients. Immunopotency (i.e. the MSC capacity to suppress the proliferation of allogenic activated T-cells) was used as the main readout of MSC function. Initial findings confirmed that atherosclerotic-MSC have impaired immunomodulatory capacity and a pro-inflammatory secretome, both contribute to the state of chronic low-grade inflammation that promotes atherosclerosis progression [5]. Moreover, we demonstrated that MSC immunopotency can indeed be enhanced by modulating inflammatory components of the MSC secretome [6]. Finally, we defined key aspects of the human atherosclerotic MSC signature: (i) morphological and structural alterations of mitochondria, (ii) mitochondrial dysfunction associated with oxidative stress, (iii) a metabolic shift toward glycolysis and reduced ATP production, (iv) a pro-inflammatory secretome, and (v) the reduced MSC ability to deal with stress was accompanied by their enhanced susceptibility to apoptosis [7]. Notably, mitochondria-targeted ROS scavengers improved the immunopotency and the survival of atherosclerotic MSC.

There are multiple potential implications of these data. First, the therapeutic effectiveness of atherosclerotic-MSC is likely compromised when compared to their non-atherosclerotic counterparts. Accordingly, only non-atherosclerotic MSC should be used in clinical trials. Second, the ability to modulate the redox state of MSC is a possible strategy to enhance the therapeutic efficacy of autologous atherosclerotic-MSCs. Third, increasing age is an established independent risk factor for the development of atherosclerosis. Notably, mitochondrial dysfunction is not only associated with aging, but also with premature or accelerated atherosclerosis [8]. Our study was not designed to address the contribution of MSC dysfunction to atherosclerosis onset or progression. However, our results strongly suggest this link, and we have set the stage to test this hypothesis in the future.
